# Cholera vaccine clinical trials: A cross-sectional analysis of clinical trials registries

**DOI:** 10.1080/21645515.2023.2261168

**Published:** 2023-09-27

**Authors:** Lindi Mathebula, Thobile Malinga, Mammekwa Mokgoro, Duduzile Ndwandwe, Charles S. Wiysonge, Glenda Gray

**Affiliations:** a Cochrane South Africa, South African Medical Research Council, Cape Town, South Africa; b Vaccine-Preventable Diseases Programme, World Health Organisation Regional Office for Africa, Brazzaville, Congo; c Office of the President and CEO, South African Medical Research Council, Cape Town, South Africa

**Keywords:** Cholera, Cholera vaccines, vaccines, clinical trials, randomised controlled trials

## Abstract

Cholera has been one of the world’s biggest public health challenges for centuries. The presence of this disease brings into focus the social determinants of health in different parts of the world. Research and development efforts to find safe and effective Cholera vaccines are critical to decreasing the disease burden from *Vibrio cholerae*. We searched the International Clinical Trials Registry Platform (ICTRP) and Cochrane Central Register of Controlled Trials (CENTRAL) on 5 March 2023. We included all registered randomized trials studying Cholera vaccines. We used Microsoft Excel to perform a descriptive analysis of the source registry, geographic distribution, recruitment status, phase of trials, and type of trial sponsor and presented the findings using tables and graphs. The search of ICTRP yielded 84 trials, and 315 trials were identified from CENTRAL. Seventy-four trials were included in the analysis. Most of the trials (66%, *n* = 49) were registered in ClinicalTrials.gov, followed by Clinical Trials Registry – India (9%, *n* = 7) and the Cuban Public Registry of Clinical Trials (8%, *n* = 6). The geographical distribution of the trials indicates that 48% (*n* = 36) of the trials were conducted in Asia, followed by 23% (*n* = 17) in North America, 15% (*n* = 11) in Africa, and 11% (*n* = 8) in Europe. Results further indicate that 81% (*n* = 60) of trials have a recruitment status “Not recruiting,” followed by 12% (*n* = 9) with a status “recruiting.” With the recent surge in Cholera cases and the limited supply of Cholera vaccines, research indicates the need for Cholera vaccine trials to ensure the availability of vaccines, especially in populations affected.

## Background

Cholera has been one of the world’s biggest public health challenges for centuries.^1^ The presence of this disease brings into focus the social determinants of health in different parts of the world. Cholera is a disease that results from infection with *Vibrio cholerae*, a facultative anaerobic motile Gram-negative curved rod with a single polar flagellum.^2^ This pathogen can cause infection in all age groups, although nursing infants may be protected by secretory antibodies in breast milk.^3,4^

The mode of transmission of *Vibrio cholerae* is via the fecal-oral route.^1,5^ The bacterium is found in water and food that has been contaminated by feces from an infected person. Shellfish, which is eaten raw or undercooked, is a common source of infection. It spreads when there is inadequate water treatment, inadequate hygiene, and poor sanitation.^6^ This can be a result of poverty where people lack access to clean water or natural disasters such as floods, cyclones, and earthquakes that can cause major disruptions to existing infrastructure and humanitarian crises which lead to the displacement of populations and overcrowding.^7,8^

Since mid-2021, the world has been facing an acute upsurge of multiple Cholera outbreaks, especially in areas that are not endemic for Cholera, with high mortality rates.^9^ World Health Organisation (WHO) for African and Eastern Mediterranean regions had 23 countries reporting Cholera outbreaks in 2021.^10^ The trend continued into 2023, with over 30 countries reporting Cholera cases or outbreaks in five WHO regions. Asia and Africa are witnessing an exponential rise in Cholera cases amid a global surge.^10^ Afghanistan, among other countries in Asia, had the highest number of recorded cases of Cholera globally as of July 2023 (23 298 cases) compared to 10 387 cases reported by July 2022.^11^ In the African continent, cases recorded in the first month of 2023 alone had already risen by more than 30% of the total caseload reached in 2022.^12^ An estimated 26 000 cases and 660 deaths have been reported as of 29 January 2023 in 10 African countries facing outbreaks since the beginning of the year.^13^ In 2022, nearly 80 000 cases and 1863 deaths were recorded from 15 affected countries.^13^ If the fast-rising trend continues, it could surpass the cases recorded in 2021, Africa’s worst year for Cholera in nearly a decade.

The average case fatality ratio is almost 3%, above the 2.3% reached in 2022 and far exceeding the acceptable level below 1%.^13^ Most new cases and deaths have been recorded in Malawi, facing its worst Cholera outbreak in two decades.^11,13^ Malawi’s neighbors Mozambique and Zambia have also recently reported cases. In East Africa, Ethiopia, Kenya, and Somalia are responding to outbreaks amid a prolonged and harsh drought that has left millions of people in dire need of humanitarian assistance. Burundi, Cameroon, the Democratic Republic of the Congo and Nigeria have also reported Cholera cases.^11,13^ The countries facing complex humanitarian crises with fragile health systems aggravated by climate change pose a challenge to outbreak response and present a risk for further spreading to other countries. Given the scale of this infectious disease, access to safe and effective vaccines to prevent Cholera are critical during outbreaks, in endemic areas, and when there is a humanitarian crisis that could lead to the spread of disease.^14^

A number of oral Cholera vaccines (OCVs) have been developed and proven safe, immunogenic, and effective.^15^ Four of these vaccines have been licensed in some countries. mORCVAX™ (killed WC plus recombinant CTB (WC/rCTB) by Crucel/SBL, Sweden) is used mainly by travelers visiting Cholera-endemic countries but has also been used in demonstration programs for public health interventions. ORC-Dukoral® (killed WC vaccine) has been modified,and technology transferred to Viet Nam where it is produced by VaBiotech. Vax® by Shantha Biotechnics was licensed in India after South-South technology transfer. And lastly, Shanchol® by EuBiologics Co., Ltd has been licensed in Korea. These four vaccines are given in two-dose regimens 2 weeks apart for Shanchol, ORC-Vax, and Euvichol and one week apart for Euvichol® and elicit protective efficacy eight days after the second dose.^15^ Dukoral® is available in many countries but is used primarily as a travel vaccine.

Another oral Cholera vaccine that is used for travelers to places with active transmission and which has been approved by the United States Food and Drug Administration (U.S. FDA) is Vaxchora.^5^ It is given as a single dose to individuals aged 2 to 64 years old. The only other licensed vaccine is a single-dose vaccine mORCVAX™ (recombinant live oral vaccine CVD103-HgR) manufactured by Crucel/Berna Biotech, Switzerland, stopped production in 2004.^15^ With the limited supply of Cholera vaccines and the recent surge in cases, there is a need for Cholera vaccine trials to ensure the availability of the vaccines, especially in populations affected.

In the United States (U.S.), a First in Human Phase 1 Ascending Dose Study of PanChol in Healthy Volunteers is currently recruiting.^16^ In South Asia, recruitment is ongoing for A Phase III, Multicenter, Observer-Blinded, Randomized, Active Controlled Trial to Evaluate Immune Non-Inferiority, Safety and Lot-to-Lot Consistency of Oral Cholera Vaccine-Simplified Compared to ShancholTM in 1 to 40 Years Old Healthy Nepalese Participants.^17^ Another vaccine, WC-BS OCV, showed 64% protection and WC without BS 56% protection against any diarrhea and both showed 100% protection against clinically significant Cholera during renewed phase 1 and phase 2 studies in Sweden, US, and Bangladesh.^18^ New and novel approaches to Cholera vaccine development are showing great potential to curb the spread of the Cholera toxin, however, research shows there are still unmet needs to be identified in the landscape of Cholera vaccination to direct research and resources to the most affected populations.^8^

Research and development efforts to find safe and effective Cholera vaccines in endemic areas are critical to decreasing the burden of disease from *Vibrio cholerae*.^7^ Clinical trials play an important role in evaluating the safety and efficacy of Cholera vaccines. This involves rigorous monitoring and surveillance to track vaccine efficacy, adverse events, and other relevant outcomes that improve Cholera prevention and treatment strategies to reduce Cholera-related morbidity and mortality, especially in resource-limited settings.^19^ Randomized Controlled Trials (RCTs) in the absence of a systematic review of synthesized evidence are the highest form of evidence to evaluate the effectiveness of medical interventions such as introduction of vaccines.^20^ RCTs conducted in regions where Cholera is endemic or prone to outbreak may provide valuable insights, close to real-world effectiveness of the Cholera vaccines in endemic areas. Thus, in this paper, we aim to identify and describe RCTs of Cholera vaccine that are ongoing, planned, or completed.

## Methods

This study is a cross-sectional analysis of registered Cholera vaccine randomized controlled trials. We conducted searches in the International Clinical Trials Registry Platform (ICTRP) and the Cochrane Central Register of Controlled Trials (CENTRAL). The ICTRP is a repository hosted by the World Health Organisation (WHO), which contains regularly updated clinical trial data from primary clinical trial registries of the WHO network. CENTRAL is a database of clinical trial records obtained from multiple sources, including PubMed, Embase, and repositories of unpublished clinical trial data such as ClinicalTrials.gov and ICTRP.

On 5 March 2023, we sought to identify randomized trials studying Cholera vaccine by searching the two databases, ICTRP and CENTRAL, using the search term “Cholera vaccine.” The search output was downloaded in two excel sheets and combined to remove duplicates. Two researchers screened the titles and summaries of the records to select eligible studies. We included all randomized trials of Cholera vaccines. All studies that were not addressing Cholera vaccine trials, non-randomized trials, were excluded and non-human trials. The rationale for choosing RCTs only is because they are designed to distribute both known and unknown confounding variables across groups and allocation of participants helps ensure that any observed differences in outcomes are more likely to be attributable to the intervention being studied rather than other external factors. While non-randomized trials might be susceptible to imbalances in baseline characteristics that could affect the accuracy of the results. We considered planned trials as those with a recruitment status “not yet recruiting” or “not recruiting;” ongoing trials as those with recruitment status “recruiting,” “enrolling participants” or “Enrolling by invitation,” “closed to recruitment, follow up continuing” or “Suspended;” and completed trials as those with recruitment status “completed,” “terminated,” or “stopped early.” We used trial identification number/registry number of ongoing trials, identified through the ICTRP, to search if these trials had a peer-reviewed publication linked to the record. Using Microsoft Excel, we performed descriptive analysis of the source registry, geographic distribution, recruitment status, phase of trials, and type of trial sponsor. We presented these findings using tables and graphs.

## Results

**Figure 1.**
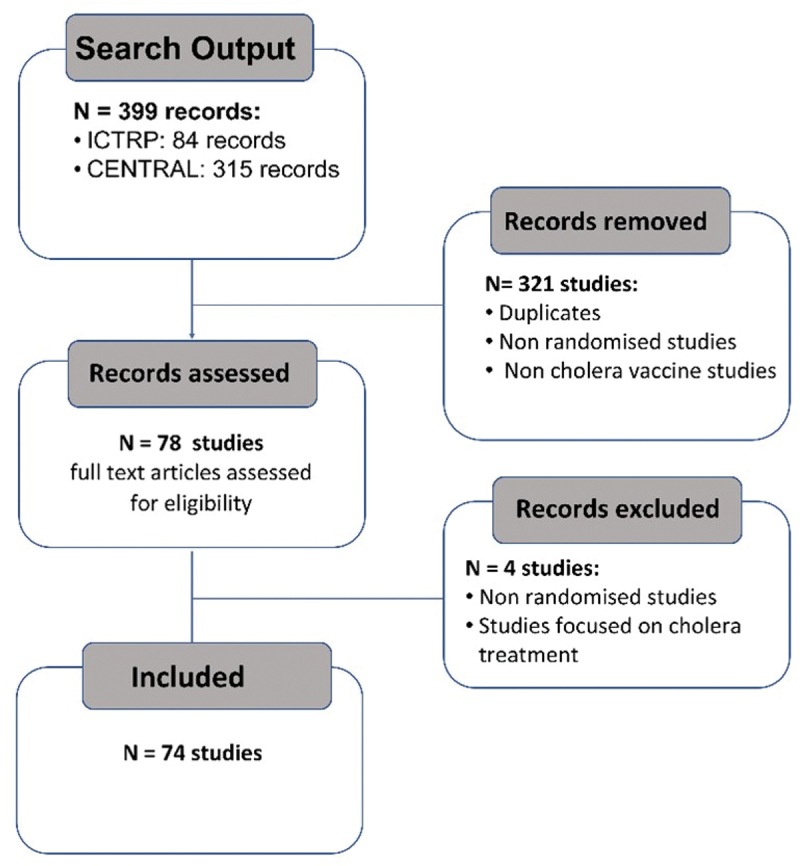
Flow diagram.

The search output yielded 84 studies identified in the ICTRP, and 315 studies identified in CENTRAL. After exclusion of duplicate and ineligible studies, we included 74 eligible trials studying Cholera vaccines in our analysis (Figure 1). Of these trials, 81% (*n* = 60) were planned trials, 15% (*n* = 11) were ongoing trials, and 4% (*n* = 3) were completed trials. The results showed that 66% (*n* = 49) of the trials were registered in ClinicalTrials.gov, followed by 9% (*n* = 7) in the Clinical Trials Registry – India (CTRI), and 8% (*n* = 6) in the Cuban Public Registry of Clinical Trials (RPCEC) registries (Table 1).

**Table 1.** Characteristics of registered Cholera vaccine trials.

	N (%)
**Trial status**	
Planned	60 (81)
Ongoing	11 (15)
Completed	3 (4)
**Registry name**	
ClinicalClinicalTrials.gov	49 (66)
CRIS	2 (3)
CTRI	7 (9)
EU Clinical Trials Register	2 (3)
ISRCTN	1 (1)
JPRN	2 (3)
PACTR	3 (4)
RPCEC	6 (8)
UMIN-CTR Clinical Trial	2 (3)
**Phase of trial**	
*Phase 0	1 (1)
Phase 1	12 (16)
Phase 1/2	2 (3)
Phase 2	20 (27)
Phase 3	16 (22)
Phase 4	11 (15)
Not reported	12 (16)
**Age of participants in trials**	
Infants	7 (9)
Preschool	24 (32)
Children	2 (3)
Adolescents	2 (3)
Adults	33 (45)
Not reported	6 (8)
**Type of primary outcome**	
Safety and immunogenicity	18 (24)
Safety and efficacy	2 (3)
*Immune response	31 (42)
Safety	12 (16)
Efficacy	6 (8)
Unclear	5 (7)
**Sponsor Type**	
Government	3 (4)
Hospital	6 (8)
Industry	16 (22)
Non-profit organization	3 (2)
Research organization	30 (41)
University	16 (22)
**Total number of trials**	**74 (100)**

CRIS (Clinical Research Information Service); CTRI (Clinical Trials Registry-India); EU (European) Clinical Trials Register, ISRCTN (International Standard Randomised Controlled Trial Number); JPRN (Japan Primary Registries Network); PACTR (Pan African Clinical Trial Registry); RPCEC (Cuban Public Registry of Clinical Trials); UMIN-CTR (University hospital Medical Information Network Clinical Trial Registry). *Phase 0: Trials that use a very small volunteer sample to test a proposed drug intervention. *Immune response: antibody response, seroconversion, seropositivity, etc.

### Geographic location of trials

**Figure 2.**
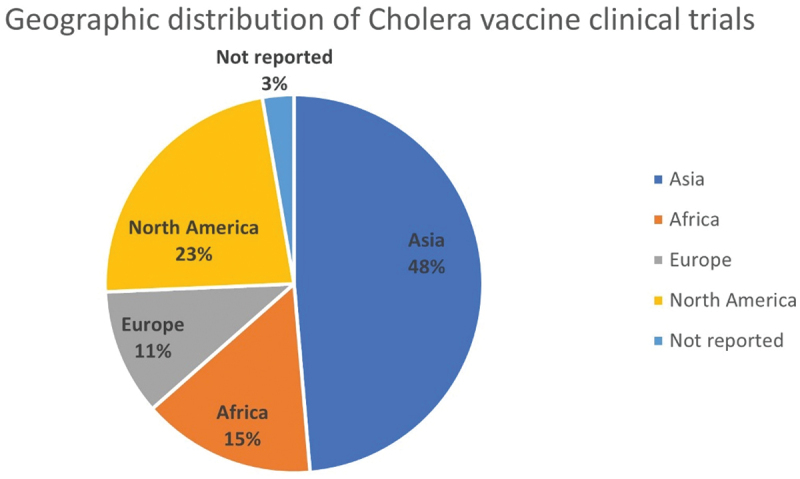
Geographical distribution of trials studying cholera vaccines.

Results on geographical distribution of the trials indicate that 48% (*n* = 36) of the trials registered in clinical trial registries were conducted in Asia, followed by 23% (*n* = 17) in North America, 15% (*n* = 11) in Africa, and 11% (8) in Europe (Figure 2). Two (3%) of the trials did not list the recruitment center.

### Type of vaccines studied by geographic location

**Figure 3.**
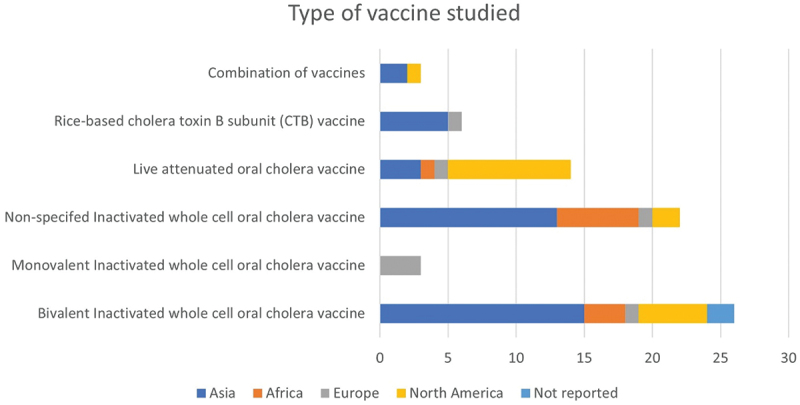
Type of vaccine studied in registered trials by continent.

In Figure 3, results showed that bivalent Inactivated whole cell oral Cholera vaccine was the most studied (35%; *n* = 26) type of vaccine in trials across the geographic location with 77% (*n* = 20) of the trials recruiting in Asia and 19% (*n* = 5) recruiting in North America. Non-specified inactivated whole cell oral Cholera vaccine is the second type of vaccine mostly studied (30%, *n* = 22), with 59% (*n* = 13) of the trials recruiting in Asia and 27% (*n* = 6) of the trials recruiting Africa. Live attenuated oral Cholera vaccine trials accounted for 19% (*n* = 14) trials across the geographic regions, with 64% (*n* = 9) recruiting in North America and 21% (*n* = 3) recruiting in Asia. Monovalent inactivated whole cell oral Cholera vaccine was studied in trials recruiting in Europe (4%; *n* = 3) trials. Eight percent (*n* = 6) trials studied the rice-based Cholera toxin B subunit (CTB) vaccine, of which 86% (*n* = 5) recruited in Asia and 14% (*n* = 1) recruited in Europe. A few trials (4%; *n* = 3) studied a combination of two vaccines, 67% (*n* = 2) recruiting in Asia and 33% (*n* = 1) recruiting in Europe.

### Phase of trials and the ages studied

Table 1 data show that majority of trials (27%; *n* = 20) registered were in phase 2 followed by 22% (*n* = 16) trials in phase 3. One trial reported phase 0 and 16% (*n* = 12) of the trials did not indicate the phase of a trial. Majority of the trials (45%; *n* = 33) studied participants in the adult age-group, this was followed by 32% (*n* = 24) participants in the preschool age group. When looking at the phase of the trials by ages, 25% (*n* = 5) trials in phase 2 were studying Cholera vaccine in infants, 30% (*n* = 6) in preschool and another 30% (*n* = 6) in adults. This was followed by 22% (*n* = 16) of trials in phase 3, of which 50% (*n* = 8) studied preschool and the other 50% (*n* = 8) studied adults. 16% (*n* = 12) of the trials were in phase 1, of which 80% (*n* = 10) studied adults only. Another 16% (*n* = 12) of the trials did not report the phase of trial, which 50% (*n* = 6) studied adults and 25% (*n* = 3) studied preschool. The most studies age group across the phases is adults with 45% (*n* = 33) trials, followed by preschool with 32% (*n* = 24) trials.

### Primary outcome of trials

Results indicates the most studied primary outcome is immune responses (42%; *n* = 31), followed by safety and immunogenicity in 18% (*n* = 24) trials and safety of the vaccine in 16% (*n* = 12) of the trials. Fewer trials (8%; *n* = 6) had efficacy as an outcome of the trial, 3% (*n* = 2) trials had safety and efficacy as an outcome. Seven percent (*n* = 5) trials listed an outcome that was unclear (Table 1).

### Recruitment status and publications

Results show that 81% (*n* = 60) of trials have a recruitment status “Not recruiting,” followed by 12% (*n* = 9) with a status “recruiting.” When exploring number of trials with publications, we found that 48% (*n* = 36) of the trials have a publication listed in the record. Of the 81% (*n* = 60) of trials that had recruitment status “Not recruiting,” 55% (*n* = 33) have a publication listed. Fewer trials (4%, *n* = 3) that have a status “Completed” had 33% (*n* = 1) publication listed. Two other trials (3%) did not list a recruitment status but indicated the status of trials as “ongoing” and had no publication listed in their records.

### Type of sponsor

**Figure 4.**
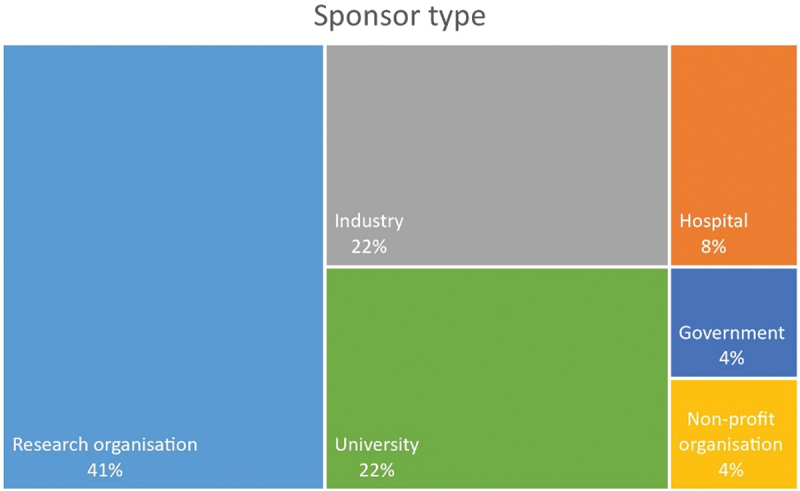
Type of sponsors registering cholera vaccine trials.

Results shows that most trials (40%; *n* = 30) trials were sponsored by research organizations, followed by 22% (*n* = 16) sponsored by industry and another 22% (*n* = 16) sponsored by university (Figure 4). Eight percent (*n* = 6) of the trials were sponsored by hospital and a few trials (4%, *n* = 3) government and nonprofit organizations each.

## Discussion

The capacity to respond to multiple outbreaks continues to be strained due to the global lack of resources, including the oral Cholera vaccine, and overstretched public health and medical personnel dealing with various disease outbreaks simultaneously. Clinical trials are ongoing to increase the toolkit of Cholera vaccines. We analyzed the vaccine clinical trials conducted globally by accessing the trial registration information on the ICTRP. Our data show that most vaccine trials were registered in clinicaltrial.gov and not available in the respective primary registries in the regions where trials are taking place. Forty-eight percent of the trials are taking place in Asia; however, only 14% of the trials are listed in primary registries of the regions of Asia. Similarly, 15% of the trials in are taking place in Africa but only 4% of the trials are registered in PACTR, the primary registry catering for African region. A study reviewing Trends in clinical trial registration in Africa showed that most trials taking place in Africa were registered in more than three registries including PACTR.^21^ Registering trials in the primary registry in the region where the trial is taking place is important to ensure that research efforts or research gaps in the region are easily identified.

Asia and Africa are leading in the number of cases reported worldwide with almost 80% of the cases reported between May 2023 and July 2023 worldwide coming from African and Asian countries.^11^ While Asia is well represented in the number of Cholera vaccine trials taking place in the continent (48%). Africa sees a surge in cases since mid-2021; however, only three Cholera vaccine trials are registered in PACTR, the only African WHO primary registry and representing only 15% of the Cholera vaccine trials globally. It is also noted that while Asian countries report a high number of cases; they have fewer Cholera-related deaths reported compared to African countries. Between 21 June and 20 July 2023, four of the five countries reporting highest death cases from Cholera were Cameroon (95 deaths), Democratic Republic of the Congo (74 deaths), Ethiopia (65 deaths), Zimbabwe (34 deaths).^11^ Zimbabwe is one country that has constantly reported a high number of cases, posing a risk of this disease spreading to many parts of Africa.^9^ The findings suggests that more Cholera vaccine trials (i.e., 37%) are conducted in places that require the least efforts to reduce the spread of the disease. Research also indicates that more efforts are made for travelers from upper middle-income countries traveling to Cholera affected areas to receive Cholera vaccines or chemoprophylaxis.^8^

The most studied type of vaccine in Asia was bivalent Inactivated whole cell oral Cholera vaccine which accounted for 77% of the trial study this vaccine type. According to research two type of bivalent Inactivated whole cell oral Cholera vaccine, mORCVAX™ and Cholvax are developed, manufactured, and only available in Vietnam and Bangladesh, respectively.^8^ There has also been mass vaccinations during Cholera outbreaks in Vietnam, Bangladesh, and India using the bivalent inactivated whole cell oral Cholera vaccine.^8^ The vaccine trials are now directing their focus toward preschool children and adults in phases 2 and 3. This shows that clinical research aims to help those most affected by Cholera. There are 4 million global cases and 143,000 deaths, with young children under 5 being the most vulnerable.^22^ When exploring the type of outcomes expected from the trials conducted, findings showed that the most studied type of outcome was immune responses such as presence of antibodies, seroconversion, and seropositivity accounting for 42% of the trials. Efficacy of the vaccine as a primary outcome was least studied by Cholera trials (8%). Research shows that OCVs have had diminished or lower efficacy in developing countries compared to developed countries.^23^ This indicates that studies on efficacy of the Cholera vaccines are crucial to determine the effect of vaccines in developing countries which are the most affected by Cholera outbreaks.

Another important finding when mapping Cholera vaccine research activity is the status of registered trials. Whether a trial is planned, ongoing or completed is important to determine the availability of results for evidence-based decisions.^24^ When exploring the recruitment status of trials, we found that of 74 trials registered, 81% had the recruitment status “not recruiting,” indicating that these were planned trials; however, 55% of these trials had a publication listed in the records. Twelve percent of the trials had a recruitment status “recruiting” indicating that these trials were ongoing, however, a publication was listed in 22% of the trial records. Only 4% of the trials had a recruitment status “completed” and these were considered completed trials where 33% of these had a publication listed in the records. The discrepancies in the data recorded suggest that trial details are not regularly updated, leading to poor quality data and a lack of transparency in reporting results.^24,25^ The development of an effective Cholera vaccine is a complex and challenging process, requiring careful navigation through a multitude of obstacles. These obstacles include the unpredictable nature of Cholera outbreaks, difficulties in recruiting participants from endemic areas, navigating regulatory clearance, and managing the ethical complexities of informed consent.^26,27^ Balancing scientific accuracy with public health urgency adds another layer of difficulty. Despite these challenges, research organizations remain committed and dedicated to overcoming them and achieving a breakthrough in Cholera vaccine development. Our finding show that research organizations account for 41% of sponsor types. Funding agencies play a crucial role in this journey, as they can provide the necessary resources to amplify momentum and catalyze progress toward this important medical goal.

The Wellcome Trust announced a funding call in early 2023 for generating evidence for decision-making on the use of the oral Cholera vaccine. This funding call aimed to generate evidence for decision-making on the use of the oral Cholera vaccine, focusing on real-world impact, and preparing for future preventative vaccination campaigns. The call also intended to fund teams to support and engage with policymakers and implementing partners responsible for preventing and controlling Cholera.^28^ Similarly, South Africa’s Biovac Institute has signed a licensing and technology transfer deal with the International Vaccine Institute (IVI) to develop and make oral Cholera vaccines for African and global markets. The partnership with nonprofit IVI headquartered in South Korea aims to boost output and reduce vaccine shortages amid a spate of global outbreaks that spurred the WHO to temporarily change its dosage regime.^29^

The project is important for Biovac as it enables drug substance manufacturing capability to be built, that is, the production of the antigen or raw material needed to manufacture actual vaccines. This is one of the remaining steps in the vaccine manufacturing value chain currently missing at Biovac and across the African vaccine manufacturing landscape. The agreement comes when Cholera outbreaks – prompted by climate change, armed conflict, and displacements – wreak havoc on fragile health systems, as observed in Pakistan, Nigeria, and Malawi. This places additional demand on the already-limited supply of Cholera vaccines globally.^27^

The limitation of this study includes that we only conducted a search on the WHO_ICTRP and CENTRAL database to obtain studies of Cholera vaccines globally, which means trial records from other clinical trial databases that are not accessible through this search platforms may have been missed. It is also noted that clinical trial records from many registries have missing fields and are not regularly updated where there could have been protocol deviations that could change the results described in the study. Furthermore, clinical trial registration is indicative of a planned, ongoing, or completed clinical trial, which may or may not be up to date and not the full trial results or publication, which could thereof paint a clear picture of Cholera vaccine trials outcomes globally.

## Conclusion

The scale of Cholera outbreaks in recent years has escalated while there has been an increasing gap between supply and demand for Cholera vaccines. A similar approach like COVID-19 rapid vaccine production is needed for Cholera as this poses a threat, especially in countries with fragile health systems and Cholera cases increasing rapidly. Furthermore, the history and current status of Cholera vaccines clinical trials demonstrate a journey of scientific innovation and collaboration sought to address a persistent global health threat. By exploring successes and difficulties of Cholera vaccine development, thus contributing to the understanding of the ongoing efforts to fight Cholera.

## Data Availability

Data are available upon reasonable request.
